# Influence of Various Irrigation Protocols on the Push-Out Bond Strength of TotalFill and AH Plus Bioceramic Sealers to Root Dentin: A Comparative *In Vitro* Study

**DOI:** 10.1055/s-0045-1806960

**Published:** 2025-04-23

**Authors:** Taher Al Omari, Alaa Dkmak, Hamza El-Farraj, Rami Haitham Albanna, Abdelmalek K. Tabnjh, Amre R. Atmeh, Jamal Aqrabawi, Hyeon-Cheol Kim, Minju Song, Sang Won Kwak

**Affiliations:** 1 Department of Conservative Dentistry, Faculty of Dentistry, Jordan University of Science and Technology, Irbid, Jordan; 2 Department of Operative, Preventive and Pediatric Dentistry, Charité-Universitätsmedizin Berlin, Berlin, Germany; 3 Department of Applied Dental Sciences, Faculty of Applied Medical Sciences Jordan University of Science and Technology, Irbid, Jordan; 4 Department of Cariology, School of Odontology, Sahlgrenska Academy, University of Gothenburg, Gothenburg, Sweden; 5 Department of Restorative Dentistry, Hamdan Bin Mohammed College of Dental Medicine (HBMCDM), Mohammed Bin Rashid University of Medicine and Health Sciences (MBRU), Dubai, United Arab Emirates; 6 Department of Restorative Dentistry, Faculty of Dentistry, University of Jordan, Amman, Jordan; 7 Department of Conservative Dentistry, School of Dentistry, Dental Research Institute, Dental and Life Science Institute, Pusan National University, Yangsan, Korea; 8 Department of Conservative Dentistry, National Health Insurance Service Ilsan Hospital, Goyang, Korea

**Keywords:** AH Plus bioceramic sealer, calcium silicate, HEDP, push-out bond strength, TotalFill

## Abstract

**Objective:**

This study aimed to compare the push-out bond strength (POBS) of TotalFill (TFB) and AH Plus bioceramic (APB) sealers with different irrigation protocols.

**Materials and Methods:**

Sixty maxillary central incisors were prepared and randomly divided into three groups ( *n* = 10) based on the final irrigation protocol. Group NC: 5.25% sodium hypochlorite (NaOCl); Group NE: 5.25% NaOCl and 17% EDTA; Group NH: 5.25% NaOCl and dual rinse HEDP (1-hydroxyethylidene-1,1-diphosphonic acid). Samples were obturated using either TFB or APB sealer only. In groups NC and NE, NaOCl was used during canal preparation, while in Group NH, NaOCl/HEDP was utilized. The teeth were then horizontally sectioned into three 3-mm thick sections at the apical, middle, and coronal levels. The POBS was performed on the root sections at a 1 mm/min speed. The failure mode was assessed using an optical microscope and a scanning electron microscope.

**Statistical analysis:**

Two-way ANOVA (analysis of variance) was used for statistical analysis to test the interaction between sealer type and irrigation solution, while an independent *t* -test was conducted to compare the means of the two sealer types at a significance level of 0.05.

**Results:**

Specimens obturated with TFB showed significantly higher POBS than APB ( *p* < 0.001). The highest bond strength was observed in the HEDP/TFB group and the lowest in the HEDP/APB group. Irrigation did not significantly influence the POBS ( *p* > 0.05). Mixed failure was most commonly observed in all groups (>65%).

**Conclusion:**

TFB sealer had improved bond strength over APB sealer, regardless of the final irrigation protocol used, which did not significantly affect the bond strength.

## Introduction

Effective root canal treatment requires thorough cleaning and shaping. Sodium hypochlorite is considered the gold standard irrigation solution in endodontics due to its disinfecting properties and ability to dissolve necrotic tissues. 1 Other irrigation materials are used to supplement the action of sodium hypochlorite, including ethylenediaminetetraacetic acid (EDTA), chlorohexidine, MTAD, and Q mix (a premixed solution of EDTA, chlorhexidine, and a detergent). 2

EDTA is a chelating agent used in conjunction with sodium hypochlorite to dissolve the inorganic root canal material and remove the smear layer created by mechanical root canal preparation. 2 It is used in the final irrigation step to enhance the cleaning efficiency of sodium hypochlorite, create open dentinal tubules, distribute the biofilm that will eventually facilitate the irrigation action, and improve the obturation. 1

A new concept of continuous chelation by weak acids has recently gained more attention. It involves using a weak chelating agent etidronic acid or 1-hydroxyethylidene-1,1-diphosphonic acid (HEDP) 3 with sodium hypochlorite. It is proposed that it will continuously remove the smear layer and enhance the efficacy of the irrigation process. 4 On the other hand, it was shown that HEDP (Dual Rinse, HEDP, Medcem Weinfelden, Switzerland) has less erosive effect on dentin surface than EDTA. 5 Also, it showed a better bioceramic sealer penetration compared to EDTA and was shown to be the most effective irrigation against *Enterococcus faecalis* . 6

EDTA, on the other hand, has a potentially harmful effect on sodium hypochlorite antibacterial and dissolving activity if used simultaneously, as EDTA depletes the action of chloride ions and can cause excessive erosion to dentinal tubules. 7 8 On the contrary, the HEDP was tested compared to sodium hypochlorite and was found to chelate Ca ^2+^ ions effectively. At the same time, the cytotoxicity and genotoxicity were dictated by the presence of free available chlorine. 9

The effects of these materials on the bonding of newer obturation materials, particularly bioceramics, are currently under investigation. 10 Bioceramics are dental materials used to fill and seal root canal systems. Understanding how irrigation solutions and chelating agents interact with bioceramics is crucial for optimizing the success of root canal treatments.

The AH Plus bioceramic sealer (APB; Dentsply Sirona, Charlotte, North Carolina, United States) is a relatively new calcium silicate-based sealer known for its rapid setting time, lower solubility, reduced film thickness, and higher radiopacity compared to the EndoSequence BC sealer (Brasseler, United States). The composition of APB includes 50 to 75% zirconium dioxide, 5 to 15% tricalcium silicate, 10 to 30% dimethyl sulfoxide, less than 0.5% lithium carbonate, and various thickening agents. 11 12

On the other hand, the TotalFill bioceramic sealer (TFB; FKG Dentaire, La Chaux-des-Fonds, Switzerland) consists of 20 to 35% tricalcium silicate, 7 to 15% dicalcium silicate, calcium phosphate, colloidal silica, 1 to 4% calcium hydroxide, and 35 to 45% zirconium oxide as a radiopacifier, along with hydroxyapatite. 12 TFB has been researched for its push-out bond strength (POBS), biocompatibility, mineralization potential, physicochemical properties, cytotoxicity, and volumetric changes. 13 14 15

This bioceramic sealer features a H _2_ O-free thickening agent that enhances flowability, making it premixed and ready for injection into root canals. 16 Various methods have been employed to test the properties of these materials, including evaluations of physicochemical characteristics, setting reactions, and POBS. 11 14 15 17

The push-out test is highly regarded as it closely simulates clinical conditions, offering greater accuracy in measuring bond strength with minimal premature failures. 18 POBS is a strong indicator of the long-term retention of dental materials within the tooth structure. Higher POBS values suggest better adhesion, which improves the durability of restorations and endodontic fillings. 19 Materials with higher POBS can better withstand the forces of mastication and thermal changes in the oral environment. This improved stress distribution helps maintain the integrity of the restoration or endodontic filling, reducing the likelihood of fractures or gaps forming between the material and tooth structure. 20 When interpreting POBS results for clinical application, practitioners should consider the specific dental material being used (e.g., different sealers or composites), the tooth structure involved (e.g., coronal vs. radicular dentin), the presence of contaminants like blood, which can significantly affect bond strength, 19 and long-term factors such as thermal cycling and masticatory loading which can impact bond strength over time. 20

To the authors' knowledge, no study has evaluated the bond strength of apical plug bioceramics (APB) to root dentin under various irrigation protocols. Therefore, this study aims to investigate the effects of different irrigation methods: sodium hypochlorite alone, sodium hypochlorite combined with EDTA, and sodium hypochlorite combined with HDEP. We will also assess their interactions with newly introduced bioceramic sealers. The null hypothesis states that neither the different bioceramic materials nor the irrigation protocols will affect the bonding strengths to root canal dentin.

## Materials and Methods

### Sample Size Calculation

The sample size calculation was based on the result of Paulson et al. 21 Accordingly, the sample size should be a minimum of eight samples in each group (power = 0.95, effect size = 0.9, significance level = 0.05)—a total of 60 teeth (10 samples in each group) with a 20% dropout. The ethical approval was granted by the IRB Committee at Jordan University of Science and Technology, Jordan (IRB ethical approval Ref-IRB/23/2017).

### Preparation of Sample

A pilot study was conducted ahead of the study with 10 teeth using the proposed methodology. The pilot study helps refine the protocol by avoiding voids in the obturated canals as no gutta-percha (GP) was used, ensuring standardization of the sample's parameters, and verifying canal lumen diameter with the POBS plugger head size compatibility. A single operator conducts all the aspects of the study. A total of 60 human maxillary central incisors were collected after extraction for periodontal reasons. Teeth were cleaned and preserved in phosphate-buffered saline in an incubator at a controlled body temperature; the solution was refreshed every week until the time of the study. Later, teeth were decorated at the level of cementoenamel junction until a standardized root length was achieved at 15 mm using a water-cooled diamond wafering blade (Horico, Berlin, Germany).

The root canal was prepared using the ProTaper Gold system (Dentsply Sirona Endodontics, Tulsa, Oklahoma, United States) up to size F5 according to manufacturer instruction to the entire working length and apically further enlarged by introducing the file 3 mm beyond the apical constriction to enlarge the canal up to ISO standard size #65, this step was made to ensure the consistency with the POBS pluggers at the apical area. The apices of all specimens were then sealed using sticky wax to prevent irrigation flow throughout the apical foramina and randomly divided into three groups ( *n* = 20) depending on the final irrigation protocol.

- *NaOCl group (Group NS)* : canals were irrigated after each sequential instrumentation with 5 mL of 5.25% NaOCl (CHLORAXID 5.25%; PPH CERKAMED, Stalowa Wola, Poland) using a 27-G side-vented needle (Vista Dental Inc., Racine, Wisconsin, United States) that was set at 2 mm short of the working length. The irrigation needle was moved upward with an amplitude of 2 mm to agitate the solution. For the final irrigation, 5 mL NaOCl (5.25%) was applied for 1 minute, followed by rinsing with 5 mL of distilled water for 1 minute.
- *NaOCl/EDTA group (Group NE)* : similar to Group NS, except for the final rinse, for which 5 mL of EDTA (17%) (i-EDTA, i-dental, UAB, Šiauliai, Lithuania) was applied for 1 minute before rinsing the canals with 5 mL of distilled water for 1 minute.
- *NaOCl/HEDP group (Group NH)* : canals were irrigated while conditioning the smear layer at the preparation stage with a capsule of 9% dual rinse HEDP mixed with 10 mL of 5.25% NaOCl (CHLORAXID, Poland) at room temperature. The solution was used with a 27-G side-vented needle; the treatment time was standard to 5 minutes for all groups; the HEDP was provided as a powder, which was mixed with NaOCl as per manufacturer's instruction for 2 minutes before use, followed by a final rinse with 5 mL distilled water for 1 minute.

In each group, samples were subdivided randomly into two groups based on the sealer type: TFB or APB. Before obturation, canals were dried using matching paper points (Dentsply Sirona Endodontics, Tulsa, Oklahoma, United States). According to their assigned groups, the sealers were applied into the canals using designated injecting tips assisted by sonic activation (EasyinSmile, Passaic, New Jersey) to disperse them. Radiographs were obtained from the proximal and labial aspects to ensure proper filling of the canals without voids. Teeth were temporarily filled with Cavit (3M ESPE, St. Paul, Minnesota, United States) and stored in an incubator at 37°C and 100% humidity for 2 weeks to allow the complete setting of the sealers.

After storage, the roots were embedded in cold-cured acrylic. Roots were then sectioned using a water-cooled precision saw IsoMet 1000 (Buehler, Lake Bluff, Illinois, United States) at three different levels: apical measure (3–6 mm above root tip), middle (6–9 mm above root tip), and coronal (9–12 mm above root tip), to obtain three slices of 3 ± 0.2 mm thickness, discarding the apical 3 mm (0–3 mm above root tip).

### Push-Out Bond Strength Measurement

For each root section, the greatest and least diameters of the canal were measured on both sides (coronal and apical), and the thickness was measured using a digital caliper (Vogal, Kevelaer, Germany). The bonded surface area was calculated using the following formula 14 :



where *D* 1: greatest canal diameter, *D* 2: least canal diameter, *µ* = 3.14, and *h* is the thickness of the root slice.

Each section was placed in the universal test machine (WDW-20, Jinan Testing Equipment IE Corporation, China). Vertical load was applied over the filling materials in an apical-coronal direction with a crosshead speed of 1 mm/min using three indenters with different sizes (1, 0.7, and 0.5 mm) corresponding to each section, coronal, middle, and apical, respectively. The maximum load at which the material was dislodged was recorded in Newton (F-Max). Accordingly, the POBS was drawn in megapascal (MPa) using the following equation:



### Examining the Mode of Failure

Root sections subjected to the push-out test were examined under 40x magnification using an optical microscope (Olympus, Tokyo, Japan) to determine the failure mode. The mode of failure was classified as follows:

- Adhesive failure between the root canal dentin wall and sealer interface.
- Cohesive failure within the sealer.
- Mixed: adhesive and cohesive modes (some material left attached to the dentin surface).

### Scanning Electron Microscopy

Two specimens from each subgroup were randomly selected for scanning electron microscopy (SEM) analysis. This step was added to help understand the result obtained from POBS. Each sample was cut in half using a metal chisel and rinsed with 99% ethanol. It was then air-dried, mounted on the metallic cylinder, and finally sputter-coated with an ultra-thin coating of gold alloy (Q150R ES sputter coater, Quorum Technologies, United Kingdom). Examination under the microscope was performed at a magnification range of 1,200 to 2,400x (Quanta FEG 450, FEI, The Netherlands).

### Statistical Analysis

The data were analyzed using IBM SPSS software Version 27, employing descriptive statistics to compute the push-out, mean, and standard deviation values. A histogram of the residuals was created to assess the data's normality and compare means across different groups. A two-way analysis of variance (ANOVA) was conducted to evaluate the effects of the sealer type and irrigation solutions. At the same time, an independent *t* -test was utilized to compare the means between the different sealers. A *p* -value of less than 0.05 was deemed statistically significant for both tests.

## Results

### Push-Out Bond Strength

The results of the POBS test of all the groups are shown in Table 1 . The two-way ANOVA test revealed that the sealer type had a highly significant effect on the POBS ( *p* < 0.05), unlike the irrigation protocol ( *p* > 0.05), with no significant interaction between the two.

**Table 1:** Push-out bond strength (MPa; mean ± SD) for the two materials tested with different irrigation on the dentine surface

	NE	NH	NS
TFB a	12.2 ± 1.83	12.4 ± 2.01	11.8 ± 1.85
APB	0.86 ± 0.31	0.56 ± 0.23	0.95 ± 0.31

Abbreviation: SD, standard deviation.

Note: Two-way ANOVA revealed that there is no significant interaction between the two parameters.

a TFB shows significantly higher push-out bond strength than the APB regardless of the irrigation protocol ( *p* < 0.05).

Furthermore, since the sealer type had a significant POBS, an independent *t* -test was done as a comparison test at 95% confidence intervals; the independent *t* -test analysis showed that TFB, regardless of irrigation protocol (12.1 ± 0.25 MPa), had a significantly higher bond strength to radicular dentine than APB (0.79 ± 0.26 MPa) ( *p* < 0.05).

Further analysis of the TFB groups using a one-way ANOVA test and post-hoc comparison revealed that both NE (12.2 ± 1.83 MPa) and NH (12.4 ± 2.01 MPa) irrigation protocols exhibited a comparable effect on the POBS. In comparison, NS (11.8 ± 1.85 MPa) was associated with a lower POBS although not significant ( *p* > 0.05). For the APB sealer, however, the highest strength was associated with the NS group (0.95 ± 0.36 MPa), although it was not significantly higher than group NE (0.86 ± 0.31 MPa) ( *p* > 0.05) and group NH (0.56 ± 0.23 MPa) ( *p* < 0.05).

Table 2 presents the POBS of both materials at different levels. The results show no consistency in the POBS values and the section level. TFB has the highest POBS at the coronal level in the NS group (14.8 ± 5.60 MPa) and the lowest in the NS group at the apical level (9.66 ± 3.09 MPa). As for the APB, the highest was in the NS group at the apical (1.65 ± 1.61 MPa) and the lowest in the NH group at the middle (0.49 ± 0.18 MPa).

**Table 2:** Mean ± SD (MPa) of the push-out bond strength for each tested material at different levels

Root section	NS		NE		NH	
	APB	TFB	APB	TFB	APB	TFB
Coronal	0.99 ± 1.16	14.8 ± 5.60	0.79 ± 0.58	9.73 ± 2.96	0.64 ± 0.43	11.6 ± 4.38
Middle	0.91 ± 1.11	10.1 ± 4.09	1.07 ± 0.87	13.7 ± 2.71	0.49 ± 0.18	13.3 ± 6.38
Apical	1.65 ± 1.61	9.66 ± 3.09	0.75 ± 0.59	14.5 ± 4.28	0.53 ± 0.28	12.3 ± 5.21

Abbreviations: APB, AH Plus Bioceramic; TFB, TotalFill Bioceramic.

### Mode of Failure

A variation in the mode of bond failure between the sealers and the radicular dentine was noticed. The percentage of each mode of failure associated with the two sealers and the different protocols of final irrigation is presented in Table 3 . Most of the samples exhibited a mixed mode of failure regardless of the irrigation protocol (>65%). Interestingly, no adhesive failures were observed in the TFB group. On the other hand, APB had few adhesive failures observed when NS or NE was used (≈30%), and the least adhesive failure (13%) was associated with group NH. Remarkably, the TFB group showed a higher rate of cohesive failures than the APB groups.

**Table 3:** Percentage of mode of failure for the materials tested from the root dentine surface treated with different irrigation protocols

Irrigation	Material	Mode of failure		
		Cohesive	Adhesive	Mixed
**NS**	**APB**	**3.3%**	**30%**	**66.6%**
	**TFB**	**20%**	**0%**	**80%**
**NE**	**APB**	**0%**	**33.3%**	**66.6%**
	**TFB**	**16.7%**	**0%**	**83.3%**
**NH**	**APB**	**3.3%**	**13.3%**	**83.3%**
	**TFB**	**26.6%**	**0%**	**73.3%**

Abbreviations: APB, AH Plus Bioceramic sealer; TFB, TotalFill Bioceramic.

### Scanning Electron Microscopic Examination

**Fig. 1:**
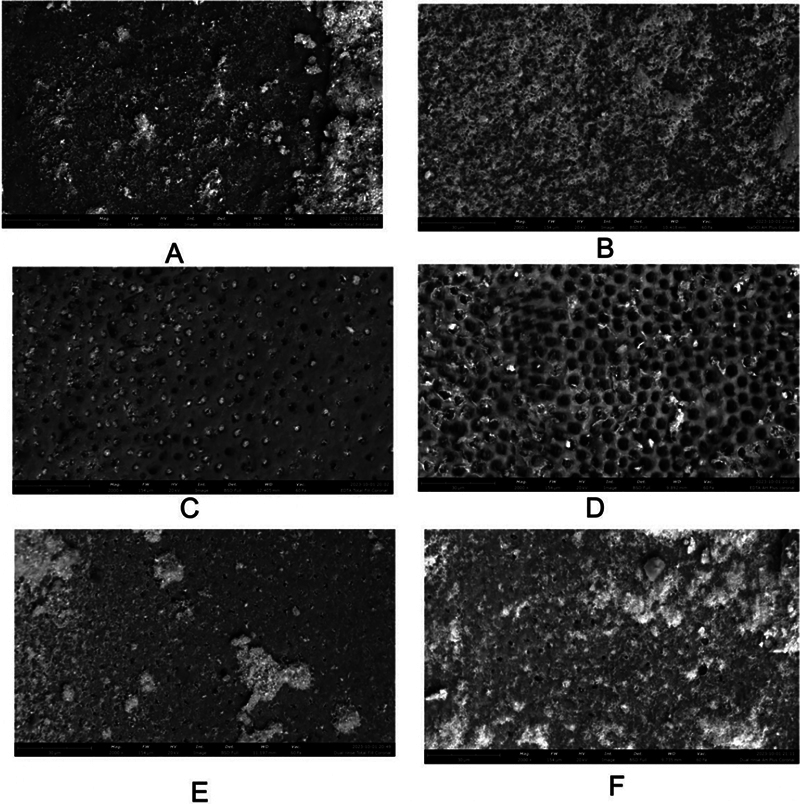
SEM of the middle part of the canal surface revealing the different root dentine surfaces treated with NaOCl/TFB ( **A** ), NaOCl/AHPB ( **B** ), NaOCl/EDTA/TFB ( **C** ), NaOCl/EDTA/AHPB ( **D** ), NaOCl/HEDP/TFB ( **E** ), and NaOCl/HEDP/AHPB ( **F** ). SEM, scanning electron microscopy.

Fig. 1 shows SEM images of the bonded dentine surface after different irrigation protocols. The images demonstrate remnant materials attached to the dentine surface in the two groups of sealers regardless of the irrigation protocol used.

Fig. 1(A, B) compares the two sealers in the NS group. The smear layer in both groups is noticeable. TFB, compared to APB, has a spot-like sealer remnant with less spread of material and less material thickness.

Fig. 1(C, D) compares the two sealers in group NE. The majority of the dentinal tubules are filled with a material that could be attributed to the TFB penetrating the tubules with remnants of the sealer adhered to the dentin surface ( Fig. 1C ). In the APB ( Fig. 1D ), no remnants of the sealer were noticeable on the dentine surface, which exhibited almost empty dentinal tubules.

Fig. 1(E, F) compares the two sealers in group NH. Interestingly, the smear layer was partially removed, and the sealers appeared to engage with it.

## Discussion

Calcium silicate cements have gained a growing interest recently due to their biocompatibility, ion release potential, and antimicrobial properties. 22 This has led to a wide range of clinical applications that include endodontic sealers. Understanding their bonding capability to root dentin is crucial because proper bonding to the root dentin will reduce fluid and microorganisms' infiltration and prevent the dislodgment of filling material to the coronal part. 23

Effective irrigation protocols are crucial for successful endodontic treatments, and recent research has provided valuable insights into optimizing these procedures. A comprehensive irrigation protocol typically involves the use of sodium hypochlorite (NaOCl) as the primary irrigant, followed by 17% EDTA for smear layer removal. 24 The sequence and volume of irrigants are critical factors, with recommendations suggesting copious amounts of NaOCl, followed by saline, EDTA, another saline rinse, and a final NaOCl rinse. 25 When tailoring their irrigation protocols, clinicians should consider case-specific factors, such as pulpal and periapical diagnoses. While there is no universally standardized protocol, clinicians should stay informed about current research and adapt their techniques based on emerging evidence to optimize endodontic outcomes. HDEP has been shown to be effective in smear layer removal, 4 effectively chelate Ca ^2+^ ions, and maintain antibacterial activity. 6 9 Also, HEPD was tested clinically and found safe and suitable for clinical use. 9

The present study investigated the effects of different irrigation protocols on the POBS of TFB and APB sealers. The results revealed that the irrigation protocol did not significantly influence the POBS of either sealer ( *p* > 0.05). For TFB, both EDTA and HEDP protocols showed slightly higher POBS values than NaOCl alone. However, these differences were not statistically significant. Conversely, for APB, the highest POBS was associated with NaOCl alone, followed by EDTA, with HEDP showing the lowest values. These findings suggest that while the choice of irrigants may subtly influence the bonding performance of bioceramic sealers, other factors, such as sealer composition and set reactions, may play a more dominant role.

HEDP is a mild chelator that increases the antimicrobial efficacy of irrigation solution. 4 It has been shown to enhance the POBS with Biodentine in partial agreement with the current study. 21 26 Our results, however, contrasted with those of Sfeir et al, 15 who reported that HEDP was associated with the least bond strength with TFB sealer. This can be explained by the different methodologies applied and the cross-section thickness of the samples.

Previous studies have explored the effect of dual rinse HEDP on the POBS of bioceramic sealers and cements. In a recent study, 27 researchers found that HEDP did not negatively affect the POBS of bioceramic cement, while a more recent study 28 reported that HEDP improved the POBS of certain bioceramic sealers. Our findings partially align with these studies, as HEDP did not significantly reduce the POBS of TFB. However, the lower POBS observed with APB when using HEDP contrasts with previous findings, highlighting the need for further investigation into the specific interactions between different bioceramic formulations and irrigation protocols.

Al-Hiyasat and Yousef 17 reported that removing the smear layer using EDTA was associated with a reduced bond strength of the TotalFill putty and MTA but not for Biodentine. This contrasts with our results. Although the same methodology was applied, this could be related to using the putty rather than sealer, which has different wettability and viscosity; on the other hand, the time storage in their study was shorter, which might have affected the calcium ion exchange with the radicular dentine and subsequently the bond strength. 29

The use of EDTA did not enhance the POBS compared to the other irrigation protocols; this is in agreement with Adham and Ali, 30 who reported no difference between the NaOCl/EDTA and HEDP. On the contrary, Sfeir et al 15 showed that NaOCl and NaOCl/EDTA irrigation protocols were associated with higher POBS when compared to HEDP when used with TFB. In the current study, NaOCl showed comparable results to HEDP and EDTA groups with TFB sealer.

Regardless of the irrigation method, each sealer had no statistically significant difference in bond strength. Therefore, the presence or absence of a smear layer after canal preparation does not significantly affect the bond strength of TFB with dentine. This can be justified by the smear layer acting as a coupling agent, enhancing the bond strength of the bioceramic sealers. 15 31 32

Compared to APB, TFB was shown to have significantly higher POBS. This could be attributed to the different compositions of these sealers. Upon hydration, calcium and hydroxyl ions release can contribute to forming tag-like structures within the dentinal tubules, which may enhance the POBS. 33 Atmeh et al 34 showed that an interfacial layer of structurally altered dentine called the “mineral infiltration zone” can form due to the alkaline etching effect of hydrated calcium silicate materials. This may explain the higher values of POBS recorded with TFB as it contains a higher percentage of silicate phases (20–35%) compared to that (5–15%) present in APB. 35 This can be supported by the findings of Souza et al, 11 in which they reported that Endosequence bioceramic sealer (a co-brand of TFB) had higher calcium ion release than APB. The latter was also found to have greater porosity related to the larger particle size. 36 On the contrary, Shieh et al 37 showed comparable POBS for both Endosequence BC and APB sealers. This difference could be related to the thin sections used (0.9 mm) in their study and the sample storage conditions.

Most of the samples exhibited mixed failure mode regardless of the irrigation protocol. It was noticed that TFB samples exhibited no adhesive failures, with most of the samples showing a mixed failure mode. This is in agreement with Dewi et al. 38 However, this result was different from Sfeir et al 15 and Shieh et al, 37 in which the authors found more cohesive failures; this can be explained by the presence of GP core material and the thin sections used in the methodology.

In this study, canals were obturated using the sealer only without utilizing GP to assess the bond strength between the sealer and dentin. Sealers present a stronger bond to dentin than to the core material (GP). Additionally, the plastic deformation of the plastic core may negatively affect the POBS. 39

Bonding of the root canal sealers to dentine was studied extensively. 15 37 38 Chemo-mechanical preparation might affect the dentinal tubule integrity, which might affect the interaction between the obturation materials and the root canal dentine. This might affect the long-term seal of the root canal sealer interface and lead to microleakage. 40 A lower bond strength might account for a higher possibility of future leakage and, subsequently, lower success of the root canal treatment. 41

However, it is crucial to acknowledge the limitations of the POBS test, which has been increasingly questioned in recent literature. 42 The assumption that higher bond strength indicates better sealer adhesion to root canal dentin walls is doubtful and lacks substantial evidence. This is particularly problematic when evaluating hydraulic materials like bioceramic sealers, which form actual chemical bonds with the dentinal surface. The POBS test, primarily a mechanical evaluation, may not accurately represent these chemical interactions. Furthermore, the test fails to account for the complex root canal anatomy, residual moisture, and dynamic oral environment, all critical factors in clinical settings.

Several limitations of this study should be acknowledged. The *in vitro* nature of the experiment may not fully replicate the clinical conditions, including the presence of residual pulp tissue, blood, or other contaminants that could affect sealer adhesion. The study also focused on several irrigation protocols and sealer types, which may not represent all clinical scenarios. Additionally, the POBS test itself, as discussed earlier, has inherent limitations in evaluating the actual bonding effectiveness of hydraulic materials.

Future research should focus on developing more clinically relevant testing methods that can better evaluate bioceramic sealers' chemical interactions, sealing ability, and long-term stability. Studies incorporating micro-computed tomography analysis of sealer penetration, long-term leakage assessments, and biocompatibility evaluations could provide a more comprehensive understanding of bioceramic sealer performance. Furthermore, investigating the effects of various irrigation protocols on the hydration and setting reactions of different bioceramic formulations could offer valuable insights into optimizing their clinical use. Lastly, long-term clinical studies comparing the outcomes of root canal treatments using different irrigation protocols and bioceramic sealers are needed to validate the clinical relevance of *in vitro* findings.

## Conclusion

APB sealer had inferior POBS compared to TFB Sealer, which might affect the long-term stability of the dentin bonding interface and treatment outcomes with APB. The irrigation protocols used in the study did not affect the POBS and the possible sealing ability of the bioceramics. Smear layer removal appears not to affect the bonding strength of the bioceramic sealers.

Based on the study results, three key conclusions can be drawn:

- TFB sealer demonstrated significantly higher POBS to radicular dentin than APB sealer, regardless of the irrigation protocol.
- The irrigation protocol did not significantly influence the POBS of either TFB or APB sealers to root canal dentin.
- Mixed failure mode was most commonly observed for both sealers across all irrigation protocols, with TFB showing a higher rate of cohesive failures than APB.
